# Effects of Salt Stress at the Booting Stage of Grain Development on Physiological Responses, Starch Properties, and Starch-Related Gene Expression in Rice (*Oryza sativa* L.)

**DOI:** 10.3390/plants14060885

**Published:** 2025-03-12

**Authors:** Parama Praphasanobol, Ratchata Chokwiwatkul, Susinya Habila, Yosita Chantawong, Teerapong Buaboocha, Luca Comai, Supachitra Chadchawan

**Affiliations:** 1 Biological Sciences Program, Faculty of Science, Chulalongkorn University, Bangkok 10330, Thailand; p.praphasanobol@gmail.com; 2 Center of Excellence in Environment and Plant Physiology, Department of Botany, Faculty of Science, Chulalongkorn University, Bangkok 10330, Thailand; ratchata.chok@gmail.com (R.C.); susinyahabila6@gmail.com (S.H.); yosita.cha@hotmail.com (Y.C.); 3 Division of Biological Science, Faculty of Science, Prince of Songkla University, Songkhla 90110, Thailand; 4 Department of Plant Science and Biotechnology, Faculty of Natural Science, University of Jos, Jos North 930003, Nigeria; 5 Omics Sciences and Bioinformatics Center, Faculty of Science, Chulalongkorn University, Bangkok 10330, Thailand; teerapong.b@chula.ac.th; 6 Center of Excellence in Molecular Crop, Department of Biochemistry, Faculty of Science, Chulalongkorn University, Bangkok 10330, Thailand; 7 Department of Plant Biology and Genome Center, University of California Davis, Davis, CA 95616, USA; lcomai@ucdavis.edu

**Keywords:** amylose, *GBSSI*, *granule-bound starch synthase I*, salt stress, yield components

## Abstract

Here, we investigated physiological responses, yield components, starch properties, and starch biosynthesis genes in five Thai rice (*Oryza sativa* L.) cultivars (SPR1, Hawm Daeng, RD43, RD69, and PTT1) with distinct starch characteristics under salt stress. Salt stress decreased flag leaf greenness (SPAD), normalized difference vegetation index (NDVI) levels, and carotenoid reflectance index 1 (CRI1) levels in all cultivars, resulting in reduced net photosynthesis, transpiration rates, and yield components across all cultivars, with Hawm Daeng and PTT1 being most susceptible. In contrast, RD69 and SPR1 were more tolerant, exhibiting recovered chlorophyll fluorescence levels and total performance index values after 3 days. Salt stress reduced apparent amylose content (AAC) and increased rapidly available glucose (RAG) levels in all cultivars. *Granule-bound starch synthase I* (*GBSSI*) expression declined the most in PTT1 and Hawm Daeng. SPAD, NDVI, CRI1, and photosynthetic parameters were correlated with *GBSSI* expression at the milky and dough stages of grain development. *GBSSI* expression levels showed little to no correlation with slowly available glucose but correlated with resistant starch levels at the booting stage of grain development. Salt stress affected yield components and rice starch quality, with variations depending on salt susceptibility, which in turn affected *GBSSI* expression levels during the milky and dough stages of grain development.

## 1. Introduction

Rice (*Oryza sativa* L.), a staple food, is a main source of carbohydrates for humans, especially in Asia [1]. Rice seed is primarily composed of starch, accounting for approximately 90% of the grain’s dry weight [2,3]. Starch consists of glucose subunits linked by glycosidic bonds and exists in two forms: amylose and amylopectin [4]. Amylose is a crucial component, with apparent amylose content (AAC) being a key determinant of rice quality, especially in terms of eating and cooking quality [5,6,7,8]. Moreover, AAC affects digestion and absorption in the small intestine, which in turn affects rapidly available glucose (RAG), slowly available glucose (SAG), and resistant starch (RS) levels [9,10]. Amylose has been positively correlated with RS and negatively correlated with RAG. Conversely, no correlation has been observed with SAG [11,12]. Rice with high amylose and RS levels, coupled with low RAG levels, offers considerable health benefits by slowing glucose release into the bloodstream.

Starch properties are affected by various factors, including environmental conditions and enzymatic activity during starch biosynthesis. Amylose production is regulated by granule-bound starch synthase I (GBSSI) activity [13,14]. Many studies have reported that *GBSSI* expression peaks during the mid-stages of grain development [15,16]. GBSSI activity progressively increases during the initial stage of rice grain development but decreases during the final stage of grain filling [17]. In addition, abiotic stresses that affect rice growth, yield, and quality present ongoing challenges to rice production. Salinity stress is a severe form of abiotic stress that limits crop production in many regions worldwide. Rice is classified as a salt-sensitive crop [18,19]. Studies have found that soils with electrical conductivities (ECs) exceeding 5 dS/m significantly reduced rice yield and quality [20,21,22,23]. Salinity causes ion toxicity and osmotic stress in plants, resulting in various physiological changes, including reduced stomatal conductivity, photosynthetic pigment degradation, inhibited growth and development, and decreased productivity [24,25]. Salinity stress, a challenge associated with climate change, impacts the growth, development, and productivity of agricultural crops [26,27] and affects approximately 20% of irrigated land worldwide [28]. With increasing climate change, sustainable food production and plant distribution across geographical areas could be affected, posing a significant threat to future food security.

Generally, rice is sensitive to salt stress during the seedling and reproductive stages of development but is relatively tolerant at the germination stage [29,30,31,32]. However, salt stress during the reproductive stage has the greatest effect on yield and grain quality [23,33]. Several studies have reported the effects of abiotic stresses on rice quality at different stages of grain development. For instance, Li et al. [34] reported that salt stress at the seedling stage caused a significant decrease in amylose content, leading to reduced grain quality. Similarly, Zhang et al. [35] showed that high-temperature stress during the booting and grain-filling stages significantly reduced both yield and amylose content. Additionally, Kumar et al. [36] reported a decrease in amylose content owing to decreased GBSSI activity during endosperm development under drought stress. Chen et al. [37] found that severe water stress during the booting, flowering, and filling stages decreased amylose content and GBSSI activity in both indica and japonica rice.

To cope with salt stress, plants have evolved various adaptive mechanisms. Salt stress initially causes osmotic stress, followed by ionic stress, resulting in osmotic and ionic homeostatic mechanisms in plants. Many genes and QTLs for salt tolerance have been identified in rice [38,39,40]. Additionally, advances in multi-omic methodologies have allowed the mechanisms of salt tolerance to be thoroughly explored [41,42]. This information can be used in breeding programs to develop salt-tolerant cultivars [43]. Liu et al. [44] summarized four major mechanisms for salt tolerance in rice: maintenance of ionic homeostasis, osmotic adjustment, ROS scavenging, and nutritional balance. Investigating these mechanisms in various rice varieties which respond differently to salt stress has significant research potential. We hypothesize that different rice cultivars with various salt-tolerance capabilities will respond differently to salt stress, leading to different effects on starch properties.

Therefore, this study aimed to investigate the effects of salinity stress at the booting stage of grain development on the productivity and quality of five Thai rice cultivars with varying starch qualities and salinity tolerances. Five Thai rice cultivars—SPR1, Hawm Daeng, RD43, RD69, and PTT1—were selected based on their starch properties. SPR1 and Hawm Daeng rice seeds possess high AAC content, while RD43 seeds have medium to low AAC content, and RD69 and PTT1 seeds have low AAC content. By analyzing leaf greenness, NDVI, CRI1 levels, and photosynthesis parameters, the most salt-susceptible cultivars and the most salt-resistant cultivars can be identified. The effects of salinity stress on *GBSSI* expression were investigated to assess changes in starch properties. The interaction between salt tolerance and starch property maintenance was demonstrated and further applications in agricultural practice and rice breeding programs are discussed.

## 2. Results

### 2.1. Different Physiological Responses to Salt Stress Were Detected in Five Rice (Oryza sativa L.) Cultivars at the Booting Stage of Development

To determine the responses to salt stress at the booting stage, we conducted a preliminary experiment to investigate the appropriate level of salt stress for salt-stress treatment. Rice plants at the booting stage were treated with 0, 75, 115, and 150 mM NaCl solution for 12 days to reach final soil ECs of 4, 6–8, 8–10, and 12–14 dS/m, respectively. At 12 days after salt treatment (DAS), rice plants recovered to normal condition (EC 4 dS/m) until harvest. Rice plants treated with 75 mM of NaCl solution did not significantly differ from the control (no NaCl treatment), while those treated with 150 mM of NaCl did not exhibit filled grains at the harvest stage (Supplementary Figure S1). Therefore, salt-stress treatment with 115 mM NaCl was selected for further experiments.

Phenotypic responses to salt stress (treated with 115 mM of NaCl) were evaluated in flag leaves at the booting stage over the following periods: after salt treatment (6 DAS and 12 DAS) and after recovery (3 DAR). Leaf greenness was determined based on SPAD levels, whereas the NDVI and carotenoid reflectance index 1 (CRI1) levels were determined using a PolyPen RP410 (Photon Systems Instruments, Drásov, Czech Republic). Plants grown under normal conditions were used as controls.

**Figure 1:**
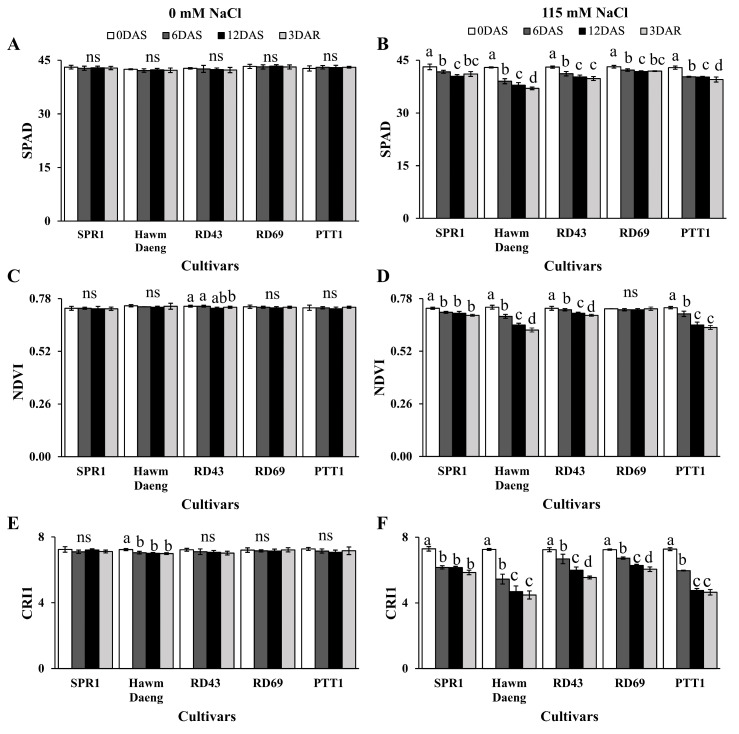
Physiological response parameters at 0, 6, and 12 days after salt treatment and 3 days after recovery. SPAD (**A**,**B**), NDVI (**C**,**D**), and CRI1 (**E**,**F**) of SPR1, Hawm Daeng, RD43, RD69, and PTT1 cultivars under normal (**A**,**C**,**E**) and salt-stress (115 mM of NaCl) (**B**,**D**,**F**) conditions. Bars represent the standard deviations of four replicates. ANOVAs were performed followed by means comparisons using DMRTs for the same cultivars. Different letters above the bars indicate statistically significant differences in means at a *p*-value < 0.05. ns represents no statistically significant difference among means.

No significant differences were observed among the five rice cultivars in any parameters or timings under control conditions (Figure 1A,C,E). Under conditions of salt stress, SPAD values for leaf greenness ranged from 42.87 to 43.13 at 0 DAS and decreased significantly with time. The cultivars Hawm Daeng and PTT1 showed the lowest SPAD values at 12 DAS. At 3 DAR, Hawm Daeng and PTT1 exhibited continuing declines in SPAD values, while SPR1, RD43, and RD69 maintained their leaf greenness (Figure 1B). These results suggest that salt stress accelerates chlorophyll degradation in the flag leaves of rice.

Salt stress reduced NDVI in all cultivars except RD69. In SPR1, NDVI decreased at 6 DAS and then remained stable until 12 DAS and 3 DAR. In contrast, NDVI declined during stress and after recovery in Hawm Daeng, RD43, and PTT1 rice. These results suggest that RD69 and SPR1 exhibited higher adaptability to salt stress compared to the other three cultivars. The lowest NDVI values were detected in Hawm Daeng and PTT1 rice, suggesting that these two cultivars had the highest salt susceptibility (Figure 1D). Moreover, CRI1 expression decreased under salt stress in all cultivars, suggesting carotenoid degradation in the flag leaves during salt stress. The lowest CRI1 values were recorded at 3 DAR in Hawm Daeng and PTT1, while the highest CRI1 value was detected in SPR1. These results suggest that SPR1 was the most salt-tolerant cultivar, whereas Hawm Daeng and PTT1 were the most susceptible to salt stress (Figure 1F).

SPAD, NDVI, and CRI1 parameters were subjected to multifactorial analysis. These parameters of the rice cultivars significantly differed between conditions and the interaction between cultivars and growing conditions significantly affected SPAD, NDVI, and CRI1 values (Supplementary Table S1).

**Figure 2:**
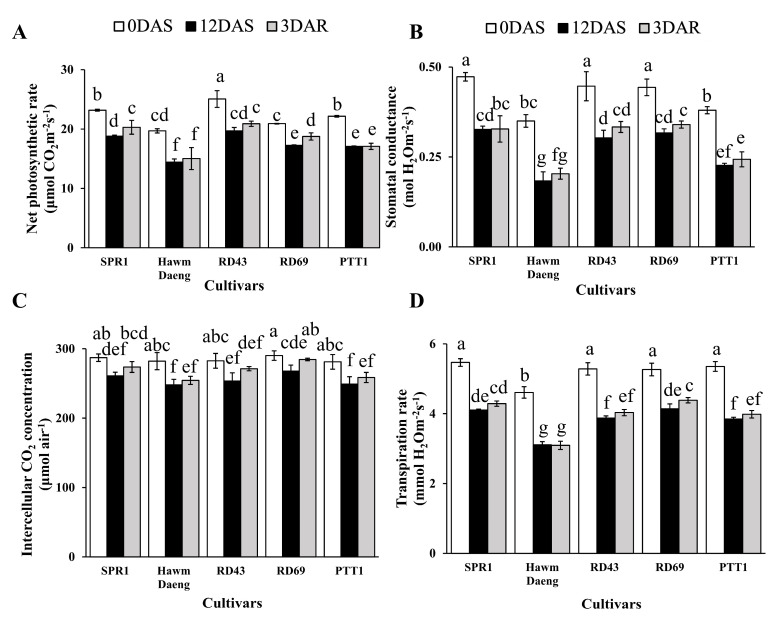
Photosynthesis parameters at 0 and 12 DAS and 3 DAR after recovery. Net photosynthesis rate (**A**), stomatal conductance (**B**), internal CO_2_ concentration (**C**), and transpiration rate (**D**) of SPR1, Hawm Daeng, RD43, RD69, and PTT1 cultivars. Bars represent the standard deviations of four replicates. ANOVAs were performed, followed by means comparisons with Duncan’s Multiple Range Tests. Different letters above the bars indicate statistically significant differences in means at a *p*-value < 0.05.

Photosynthetic parameters, including net photosynthetic rate (Figure 2A), stomatal conductance (Figure 2B), intercellular CO_2_ concentration (Figure 2C), and transpiration rate (Figure 2D), were determined in flag leaves at the onset of salt-stress treatment, 6 DAS, and 12 DAS to evaluate the effects of salt stress on photosynthetic activity in flag leaves, given their crucial role in seed production. The multifactorial analysis was performed as shown in Supplementary Table S1. The responses of these photosynthesis parameters in all cultivars were significantly different. The salt-stress condition significantly affected maximum photosynthesis rate (*P_n_*) and transpiration rate (*E*), but not stomatal conductance (*g_s_*) and internal CO_2_ concentration (*C_i_*). However, the interactions between cultivars and growing conditions were significantly different (Supplementary Table S1). This information confirmed that the cultivars used in this study had different responses in photosynthesis parameters to salt stress.

Salt stress led to a decrease in net photosynthetic rate in all cultivars at 12 DAS. However, significant recovery in the net photosynthesis rate was detected only in SPR1 and RD69 at 3 DAR (Figure 2A), suggesting that these two cultivars are more adaptable to salt stress. Salt stress had a greater impact on stomatal conductance, with a reduction of approximately 40% in Hawm Daeng, while RD69 showed a 25% reduction (Figure 2B). Stomatal conductance tended to increase post-recovery. A similar pattern was observed for intercellular CO_2_ concentration (Figure 2C). Salt stress also led to a reduction in the transpiration rate of all cultivars at 12 DAS. However, only RD69 showed a significant increase in transpiration rate at 3 DAR (Figure 2D).

**Figure 3:**
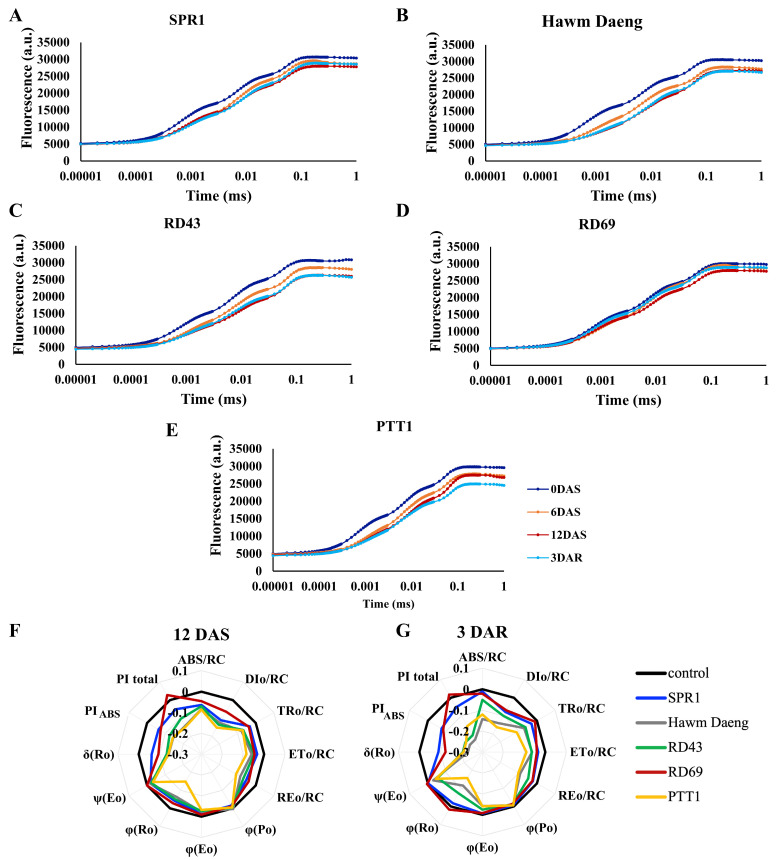
Light reaction process parameters at 0, 6, and 12 days after salt treatment and 3 days after recovery. OJIP parameters of the SPR1 (**A**), Hawm Daeng (**B**), RD43 (**C**), RD69 (**D**), and PTT1 (**E**) cultivars. Spider chart of photosynthetic parameters on 12 DAS (**F**) and 3 DAR (**G**). All experiments were performed in quadruplicate. The parameters were as follows [45]: φPo—maximum quantum yield for primary photochemistry; φEo—quantum yield of the electron transport flux from Q_A_ to Q_B_; φRo—quantum yield for reduction of end electron acceptors at the PSI acceptor side; ψEo—efficiency/probability of transfer of a trapped electron in PSII from Q_A_ to Q_B_; δRo—efficiency/probability of transfer of an electron from Q_B_ to a PSI acceptor; ABS/RC—absorbed photon flux per reaction center (RC); TRo/RC—trapping per RC; ETo/RC—electron transport flux per RC; REo/RC—electron flux-reducing end electron acceptors at PSI acceptor side per RC; DIo/RC—dissipation as heat and fluorescence per RC; PI_ABS_—performance index on the absorption basis; PI_total_—total performance index up to the PSI end electron acceptors.

To investigate the effects of salinity on light capture activity during the light reaction, the OJIP curve was monitored in all five cultivars under salt stress at 0, 6, and 12 DAS and 3 DAR. Although OJIP curves were similar across all cultivars, declines in fluorescence levels were observed after salt stress (Figure 3), suggesting lower light absorption. The multifactorial analysis revealed that all cultivars tested showed significant differences in all light reaction parameters detected by the chlorophyll fluorescence meter Pocket PEA (Hansatech Instruments Ltd., King’s Lynn, UK) (Supplementary Table S1). RD43 showed the greatest reduction in F_m_, whereas RD69 had the least reduction in F_m_ at 12 DAS, suggesting that the PSII of RD43 was more severely affected, while that of RD69 was the least affected by salt stress. At 3 DAR (blue line in Figure 3), higher fluorescence quantum yields at “P” of the OJIP curves were detected in SPR1 and RD69 (Figure 3A,D), suggesting enhanced recovery of light absorption in the photosystems of these two cultivars. Conversely, PTT1 exhibited lower fluorescence quantum yield, suggesting more extensive damage to the light absorption apparatus at post-recovery (Figure 3E). Hawm Daeng and RD43 (Figure 3B,C) showed similar fluorescence quantum yields at 3 DAR as at 12 DAS, indicating the inability of the photosystems to recover.

Additional light absorption parameters were investigated at 12 DAS (Figure 3F) and 3 DAR (Figure 3G) compared to non-stressed plant controls. At 12 DAS, the electron flux of all cultivars was affected, with the least effects observed in RD69, owing to its ability to maintain a total performance index (PI_total_) similar to that of the control. PTT1 had the lowest quantum yield for the reduction of end electron acceptors at PSI (φRo), suggesting that this cultivar was most susceptible to salt stress. At 3 DAR, declines in quantum yields ((φRo) and performance indexes were observed in RD43, Hawm Daeng, and PTT1 (Figure 3G).

### 2.2. Effect of Salt Stress on Yield Components of Rice

At the booting stage, salt stress showed no effect on the tiller number per plant, panicle number per plant, or panicle length across all rice cultivars (Table 1; Supplementary Table S1). However, salt stress increased the number of unfilled grains per panicle and caused significant decreases in the number of filled grains per panicle, total seeds per plant, seed weight per plant, and 1000-seed weight. The most pronounced reductions of more than 50% in filled grains per panicle and total seeds per plant were observed in the PTT1 and Hawm Daeng cultivars. In contrast, RD69 exhibited only a 27% reduction in filled grains per panicle. SPR1 and RD43 exhibited decreases of approximately 40% in total seeds per plant, whereas RD69 exhibited a decrease of 31% in total seeds per plant (Table 1). These results underscore the negative effects of salt stress on rice yield components, with PTT1 exhibiting the highest susceptibility and RD69 demonstrating the highest tolerance. Phenotypic responses to salt stress were evaluated in flag leaves at the booting stage.

### 2.3. Effects of Salt Stress on the Starch Properties of Rice

**Figure 4:**
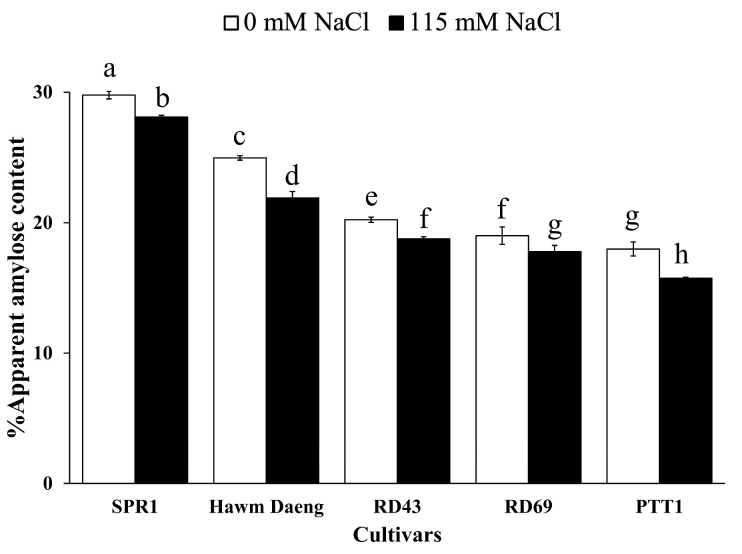
Assessment of AAC (%) in milled rice (*Oryza sativa* L.) samples from different cultivars under control and salt-stress conditions. Each value represents the mean ± SD (n = 4), with bars indicating the standard deviation of four replicates. ANOVAs were performed, followed by means comparison using DMRTs. Different letters above the bars indicate statistically significant differences in means at a *p*-value < 0.05.

AAC is important for determining the eating quality of rice. In this study, we investigated the AACs of five rice cultivars under both normal and salt stress conditions. The results showed that the AAC of rice decreased significantly under salt stress conditions. The AACs of SPR1, Hawm Daeng, RD43, RD69, and PTT1 cultivars decreased by 5.62%, 12.30%, 7.28%, 6.39%, and 12.46%, respectively, compared to the control (Figure 4).

**Figure 5:**
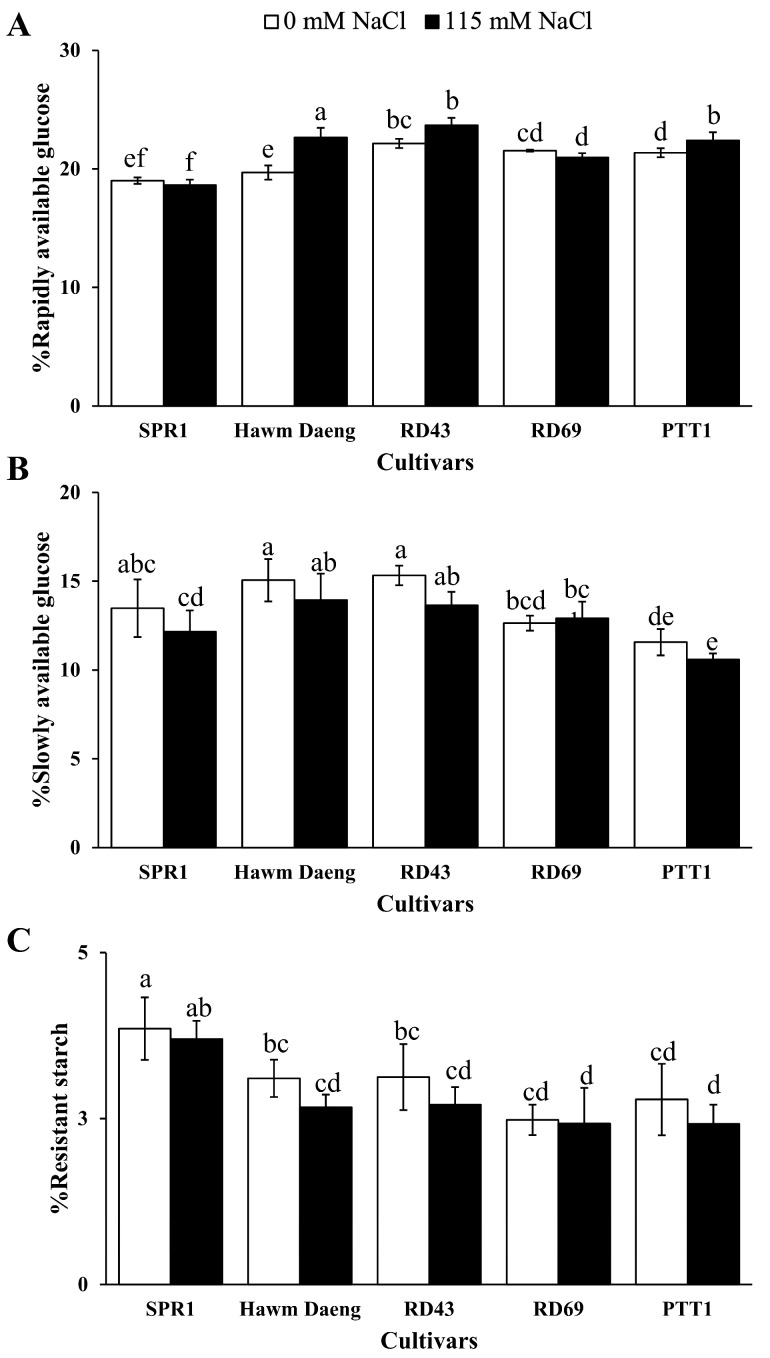
Starch digestibility (%) of milled rice samples obtained from different cultivars under control and salt-stress conditions. Parameters measured included RAG (**A**), SAG (**B**), and RS (**C**). Each value represents mean ± SD values (n = 4). Bars represent the standard deviations of four replicates. ANOVAs followed by DMRTs were performed to compare mean values. Statistically significant differences among means are indicated by different letters above the bars at a *p*-value < 0.05.

Salt stress significantly increased RAG levels in Hawm Daeng and PTT1 but did not significantly affect RAG levels in SPR1, RD43, and RD69 (Figure 5A). In contrast, salt stress slowly decreased SAG levels, with RD69 showing the least change in response to salt stress (Figure 5B). Similar results were observed in RS (Figure 5C).

### 2.4. Expression Analysis of Gene Regulating Starch Properties

Granule-bound starch synthase I (GBSSI), a key enzyme responsible for amylose synthesis in endosperm, regulates amylose content and is associated with RS content in rice. Based on a genome-wide association study (GWAS) in the Thai rice population, Praphasanobol et al. [46] hypothesized that *GBSSI* could regulate AAC and RS content in the Thai rice population.

**Figure 6:**
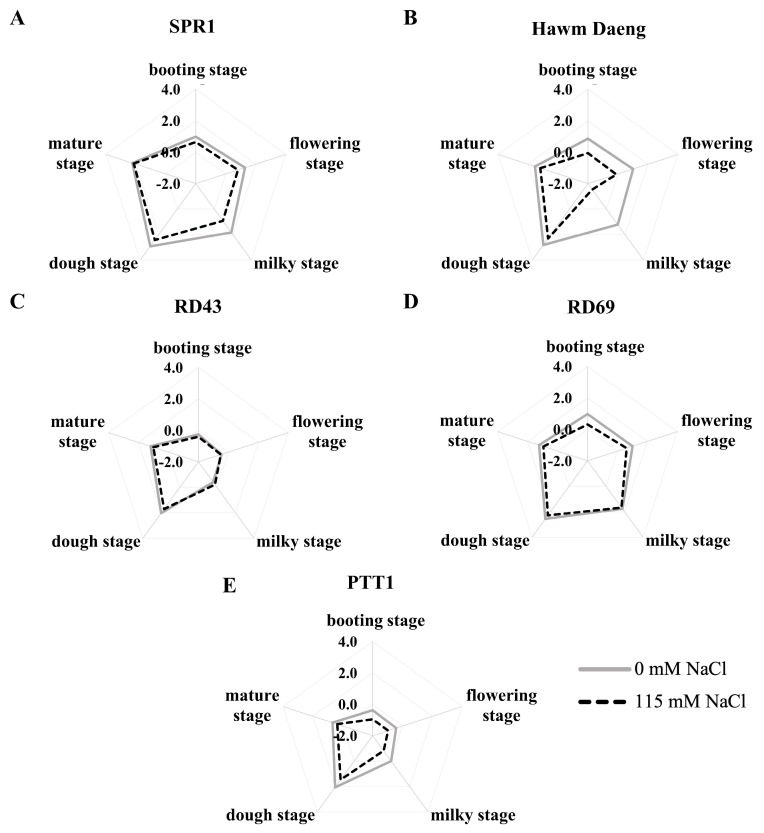
Expression of *GBSSI* at different stages of grain development in SPR1 (**A**), Hawm Daeng (**B**), RD43 (**C**), RD69 (**D**), and PTT1 (**E**) cultivars under control and salt-stress conditions. Expression levels were determined via qRT-PCR using *EF1a* as an internal control. Each value represents the mean ± SD value (n = 4). Radar charts were used to compare expression levels between developing seeds from control and salt stress-treated plants.

In the present study, the highest *GBSSI* expression was detected at the dough stage of development in plants under both normal and salt-stress conditions. Salt stress at the booting stage resulted in a decrease in *GBSSI* expression in all cultivars except RD43. Moreover, in Hawm Daeng and PTT1, a greater reduction in *GBSSI* was observed at the milky stage (Figure 6).

**Figure 7:**
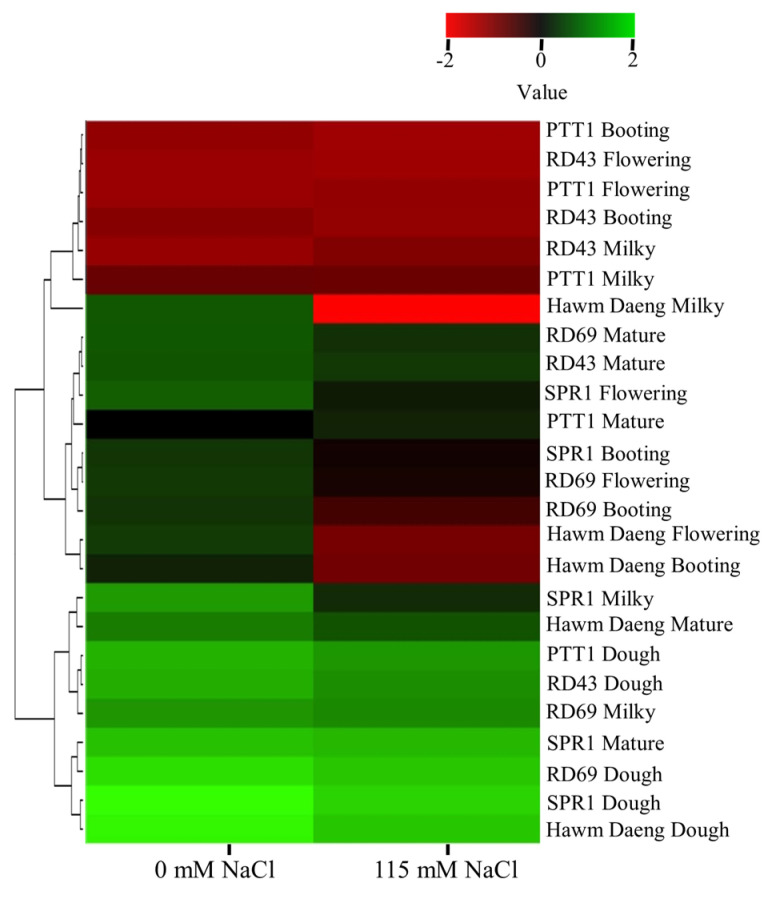
Heatmap comparison of *GBSSI* expression in developing seeds of 5 cultivars (SPR1, Hawm Daeng, RD43, RD69, and PTT1) at the booting, flowering, milky, dough, and mature stages of development.

The heatmap of gene expression was plotted as shown in Figure 7. Remarkable changes in *GBSSI* expression were detected in the Hawm Daeng cultivar, especially at the milky stage. The reduction in *GBSSI* expression in the Hawm Daeng cultivar started in the booting and flowering stages. This pattern of expression was different from those of the other cultivar. It is worth mentioning that *GBSSI* expression in PTT1 and RD43 in the booting and flowering stages was lower than *GBSSI* expression in other cultivars and salt stress exhibited slight effects on the expression of this gene in these two cultivars (Figure 7).

## 3. Discussion

The evaluation of salt stress responses through leaf greenness (determined by SPAD), NDVI, CRI1, and photosynthetic parameters revealed varying levels of salt tolerance among the five rice (*Oryza sativa* L.) cultivars. Salt stress led to a reduction in photosynthetic ability, as reported in other studies [47,48]. The reduction in the net photosynthetic rate could be attributed to diminished stomatal conductance [49,50]. Moreover, chlorophyll degradation, indicated by lower SPAD values, likely contributed to the reduced photosynthesis rate. These results are similar to those of previous studies that reported significant declines in photosynthetic pigments under salt stress [25,51,52].

The PTT1 and Hawm Daeng cultivars showed greater reductions in key parameters than the other cultivars, which indicated the higher salt susceptibility of these two cultivars (Figure 1 and Figure 2). In contrast, SPR1, RD43, and RD69 were less affected by salt stress. However, SPR1 and RD69 showed better recovery of net photosynthesis rates than RD43 (Figure 2A). This was supported by the ability of the light-harvesting apparatus to help SPR1 (Figure 3A) and RD69 (Figure 3D) recover, as observed from the OJIP curve for chlorophyll fluorescence. RD69 particularly maintained its PI_total_ during salt stress and post-recovery (Figure 3F and G, respectively). Moreover, salt-induced reductions in photon flux absorption (ABS/RC), excitation flux trapping (TRo/RC), electron transport flux (ETo/RC), and heat and fluorescence dissipation (DIo/RC) in the Hawm Daeng (Figure 3B) and PTT1 cultivars (Figure 3E) indicate that salt stress may result in less active RCs. These changes could lead to lower PI_ABS_ and PI_total_ values because energy could not be used efficiently for photochemical reactions during stress and afterwards in these two cultivars.

The reduction in photosynthesis in flag leaves resulted in a decrease in the yield components across all cultivars (Table 1). Flag leaves are major source organs, providing more than half of the nutrients for seed development [53]; therefore, their impairment under salt stress could result in a reduction in yield components, as observed in this study. These findings are consistent with those of previous studies [23,34,54,55,56].

**Figure 8:**
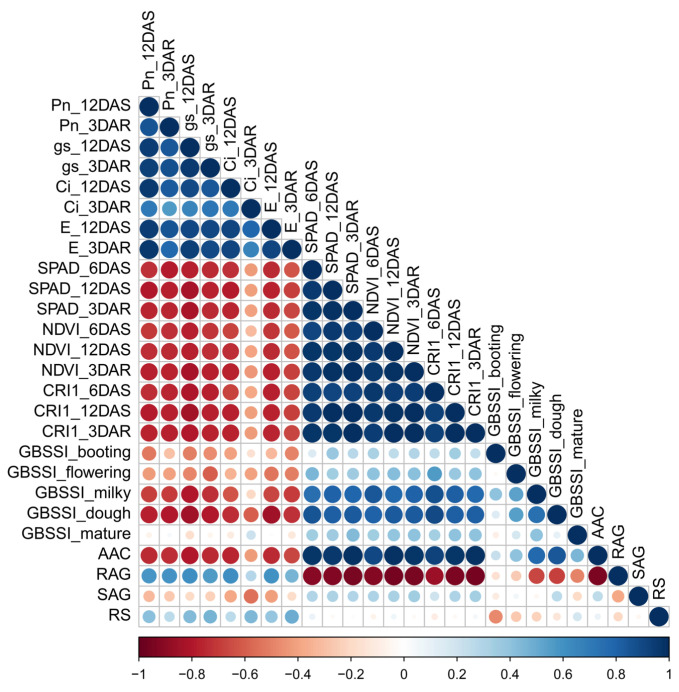
Correlation of photosynthesis parameters (including SPAD, NDVI, and CRI1) during salt-stress treatment at various seed developmental stages with *GBSSI* expression and starch properties, such as AAC, RAG, SAG, and RS in seeds.

Salt stress led to a reduction in yield components and changes in starch properties, resulting in a decline in AAC and increase in RAG. The correlation between photosynthetic capacity and starch properties was determined by measuring AAC, RAG, SAG, and RS, as shown in Figure 8. *GBSSI* expression at the dough stage was highly negatively correlated with photosynthetic parameters and positively correlated with the SPAD, NDVI, and CRI1 values of flag leaves during stress and recovery. *GBSSI* expression was strongly correlated with AAC and RAG in seeds. In contrast, *GBSSI* expression at all seed development stages showed low or no correlation with SAG, whereas a moderate correlation was observed between *GBSSI* expression at the booting stage and RS (Figure 8). The greater sensitivity to salt stress shown by the higher reduction in photosynthetic pigment content (chlorophyll and carotenoid content) (Figure 1), net photosynthetic rate (Figure 2A), and efficiency of photosystems (Figure 3), leading to a greater reduction in *GBSSI* expression, suggests that plants with more sensitivity to salt stress allow more osmotic and ionic stress to reach their flag leaves, which might be responsible for the stronger effect on *GBSSI* expression. However, *GBSSI* expression was correlated with AAC and RAG levels but not with SAG levels, raising questions as to how these relationships hold across different cultivars, particularly given the variability observed in AAC and RAG levels under conditions of stress.

Several studies have reported that AAC is positively correlated with RS content but negatively correlated with RAG [57,58,59], which agrees with our results. The decrease in AAC owing to salt stress was previously reported by Li et al. [34]. In this study, we showed that the changes in AAC and RAG levels under conditions of salt stress could be attributed to changes in *GBSSI* expression at the dough stage of seed development. Our study demonstrated that the milky and dough stages are critical for amylose synthesis. Thus, these findings demonstrate that salt stress reduces AAC by downregulating *GBSSI* under salt-stress conditions.

Our results are similar to those of previous studies showing high *GBSSI* expression levels in the mid-stage of grain development in rice [15,16]. This suggests that the milky and dough stages are critical for amylose synthesis. Furthermore, abiotic stresses have been reported to decrease the AAC in rice grains by decreasing the expression of *Wx*, a gene encoding the GBSSI enzyme, during the early and middle stages of rice grain filling [36,37,60], similar to the results of our study. These results suggest that salinity stress decreases *GBSSI* expression, potentially disrupting starch biosynthesis and amylose accumulation in rice grains. Based on our study, it is suggested that salt-stress adaptation is well correlated with the maintenance of starch properties. Therefore, genetic engineering or gene editing, including the conventional breeding program for salt tolerance, should be the key for creating cultivars with stable starch properties under climate-change conditions.

In terms of agricultural practices, these findings imply that salt stress can affect starch properties, especially in rice plants more susceptible to salt stress, leading to lower AAC content and higher RAG. Lower AAC results in a softer texture of cooked rice, which is preferred by most consumers. However, salt stress can affect yield. From a health perspective, consuming rice with lower RAG will provide more benefits. Therefore, under conditions of salt stress due to climate change, rice cultivars more resistant to salt stress should be chosen to prevent increases in RAG. Otherwise, agricultural management practices focused on preventing salt stress during the booting stage should be considered to maintain the quantity and quality of rice seeds and starch properties.

## 4. Materials and Methods

### 4.1. Plant Materials

Five rice (*Oryza sativa* L.) cultivars—Supanburi 1 (SPR1), Hawm Daeng Photoperiod insensitive (Hawm Daeng), RD43, Pathumthani 1 (PTT1), and Tab Tim Chumpae or RD69—were provided by the Pathumthani Rice Research Center, Rice Department, Ministry of Agriculture and Cooperatives. These cultivars were selected based on their starch properties, as discussed in Praphasanobol et al. [46]. SPR1 and Hawm Daeng exhibit high AAC, RD43 exhibits medium-to-low AAC, and RD69 and PTT1 exhibit low AAC. RD43 and SPR1 originated from Suphan Buri province, Central Thailand; PTT1 originated from Pathum Thani province, Central Thailand; and RD69 originated from Khon Kaen province, Northeast Thailand. There is no information about the origin location of Hawm Daeng.

### 4.2. Growth Conditions and Experimental Design

Rice seeds were germinated (until the roots were visible) in plastic cups containing distilled water for five days. Each seedling was subsequently transplanted into 8 kg of clay soil in a 30 cm pot. The soil was fertilized appropriately and maintained until harvesting. The experiment was performed using a randomized complete block design (RCBD) with four replicates (three plants per replicate) in a greenhouse at the Faculty of Science, Chulalongkorn University, Thailand, to evaluate different responses to salt stress.

For salt-stress treatment at the booting stage, water was drained before exposure to salt stress. Plants were treated with a 115 mM NaCl (Srichand United Dispensary, Bangkok, Thailand) solution to simulate salt-stress conditions, characterized by soil salinity with an EC value of 8–10 dS/m. The EC value was monitored daily. Control plants were grown under normal conditions and treated with water. The conditions were maintained for 12 days. Soil samples were collected to determine salinity levels by measuring the EC, as reported by Watling [61]. After 12 days, the plants were allowed to recover from salt stress by draining the NaCl solution and washing the soil with tap water until the soil EC reached 4 dS/m.

An analysis of variance was performed to detect the differences among means of each parameter, and Duncan’s Multiple Range Tests were used to detect significant differences between each mean at *p*-value < 0.05 using IBM SPSS 22.0 statistical software. All the results are presented as mean ± standard deviation from the mean.

### 4.3. Measurement of Physiological Parameters

To evaluate the physiological responses of treated plants, the photosynthesis responses of flag leaves were determined at 0 and 12 DAS and 3 days after recovery (DAR). A Gas Analysis System, LI-6400 (LI-COR Environmental, Lincoln, NE, USA) with a light intensity of 1200 μmol/m^2^/s^1^ and carbon dioxide (CO_2_) concentration of 380 μmol/mol was used to assess the physiological parameters, including the net photosynthetic rate (*P_n_*), stomatal conductance (*g_s_*), transpiration rate (E), and intercellular CO_2_ concentration (*C_i_*). These parameters were measured on three positions of the flag leaf of the main tiller on 0 and 12 DAS and 3 DAR in both conditions. Chlorophyll fluorescence was measured using a Pocket PEA (Hansatech Instruments Ltd., King’s Lynn, UK), according to the manufacturer’s instructions. Leaf greenness was determined using SPAD and the NDVI. Carotenoid content and the carotenoid reflectance index 1 (CRI1) were determined using PolyPen RP 400 UVIS equipment at 0, 6, and 12 DAS, and 3 DAR.

### 4.4. Measurement of Yield Components

Yield components, including the number of tillers per plant, number of panicles per plant, panicle length (cm), number of filled and unfilled grains per panicle, seed number per plant, seed weight per plant, and 1000–grain weight, were assessed after harvest under both conditions.

### 4.5. Measurement of Starch Properties

Seeds under both conditions were collected for AAC and digestible starch measurements, with all assays conducted in triplicate. AAC was determined via colorimetric analysis of the amylose-iodine complex according to the modified method reported by Juliano [62] and Praphasanobol et al. [46]. Levels of digestible starch, including RAG, SAG, total glucose (TG), and RS were determined according to the modified methods reported by Englyst et al. [9] and Praphasanobol et al. [46]. Glucose content was measured using a Megazyme kit (Megazyme International Ireland Ltd., Bray, Ireland) and converted to starch by multiplying it by 0.9. The starch fractions were calculated as follows:

RAG = G_20_,

SAG = G_120_ − G_20_,

RS = (TG − G_120_) × 0.9.

### 4.6. Gene Expression Analysis

*GBSSI* (*LOC_Os06g04200*), identified as a regulator of starch properties in a Thai rice population by a genome-wide association study (GWAS) [46], was selected to assess the effect of salinity stress on gene expression. Developing rice grains were collected at the booting, flowering, milky, dough, and mature stages to evaluate gene expression. Three biological replicates were used in the experiment. Total RNA was extracted from seed samples using the method described by Vennapusa et al. [63]. cDNA synthesis was performed using the cDNA Synthesis Kit for RT-PCR (iScript, Bio-Rad, Hercules, CA, USA) in accordance with the manufacturer’s instructions. Quantitative real-time polymerization chain reaction (qRT-PCR) was performed to measure *GBSSI* expression in developing rice grains using the Luna qPCR SuperMix (M3003L, New England Biolabs, Ipswich, MA, USA) on a Bio-Rad CFX–96 machine system. The reaction conditions were as follows: 94 °C for 30 s, followed by 40 cycles at 94 °C for 5 s, 60 °C for 15 s, and a melt curve analysis at temperatures ranging from 65 °C to 95 °C. Fold change was evaluated by using *EF1α* as an internal control, as per the method proposed by Pfaffl [64]. Primer sequences are listed in Supplementary Table S2. All reactions were performed in independent biological and technical triplicates.

### 4.7. Experimental Design and Statistical Analysis

Experiments were conducted using an RCBD with four replicates, each consisting of three plants. Analysis of variance (ANOVA) was used to evaluate the data for each parameter. Means were compared using Duncan’s Multiple Range Tests (DMRTs) with IBM SPSS Statistics software version 29.0.1, and a *p*-value < 0.05 was considered significant.

## 5. Conclusions

The experimental results indicate that salt stress at the booting stage of rice (*Oryza sativa* L.) adversely affected growth and development and led to a reduction in flag leaf greenness, NDVI, CRI1, photosynthesis abilities, and rice yield components. Based on this research, SPR1 was the most salt-tolerant cultivar, and its seed starch properties showed the fewest effects from salt stress, while Hawm Daeng was the most salt-susceptible cultivar and showed the most effects on changes in its seed starch properties. This demonstrated that salinity was associated with changes in starch properties, especially reduction in AAC and increase in RAG levels, owing to reduced *GBSSI* expression under salt-stress conditions. These findings provide a better understanding of the relationship between salt stress and starch properties during the booting stage.

In conclusion, in rice farming practice in salinity affected areas, salt-tolerant cultivars should be selected, not only due to their higher yield, but also for maintaining rice starch properties. Moreover, salt stress should be avoided by agricultural practice, especially at the booting stage, to maintain the quality of starch or AAC and RAG contents.

## Figures and Tables

**Table 1:** Yield components of five rice (*Oryza sativa* L.) cultivars under normal and salt stress conditions.

Yield Component	Condition	Rice Cultivar				
		SPR1	Hawm Daeng	RD43	RD69	PTT1
Tiller number per plant	0 mM NaCl	14.69 ± 1.40 ^ab^	14.67 ± 1.12 ^ab^	15.42 ± 0.83 ^a^	12.58 ± 0.87 ^b^	14.58 ± 1.66 ^ab^
	115 mM NaCl	12.58 ± 1.53 ^b^	15.67 ± 0.86 ^a^	15.92 ± 1.03 ^a^	12.58 ± 2.13 ^b^	14.67 ± 1.83 ^ab^
Panicle number per plant	0 mM NaCl	13.75 ± 1.10 ^a^	14.42 ± 1.32 ^a^	14.25 ± 1.52 ^a^	10.42 ± 0.50 ^c^	14.42 ± 1.45 ^a^
	115 mM NaCl	12.83 ± 1.14 ^a^	15.00 ± 0.82 ^a^	13.92 ± 1.07 ^a^	9.84 ± 0.69 ^c^	13.58 ± 1.10 ^a^
Panicle length (cm)	0 mM NaCl	26.99 ± 0.58 ^a^	25.70 ± 1.17 ^bc^	24.56 ± 0.70 ^c^	26.33 ± 0.39 ^ab^	26.50 ± 1.05 ^ab^
	115 mM NaCl	26.56 ± 0.57 ^ab^	25.59 ± 0.82 ^bc^	25.30 ± 0.25 ^bc^	27.09 ± 1.41 ^a^	25.33 ± 0.52 ^bc^
Filled grains per panicle	0 mM NaCl	116.50 ± 15.80 ^b^	112.20 ± 12.80 ^b^	107.10 ± 5.37 ^b^	149.17 ± 8.02 ^a^	114.24 ± 11.03 ^b^
	115 mM NaCl	75.14 ± 4.32 ^c^	50.59 ± 2.44 ^e^	66.16 ± 4.15 ^cd^	108.86 ± 8.85 ^b^	55.56 ± 8.13 ^de^
Unfilled grains per panicle	0 mM NaCl	25.81 ± 1.83 ^def^	24.84 ± 3.17 ^ef^	21.81 ± 2.79 ^f^	23.20 ± 2.51 ^f^	29.06 ± 4.76 ^de^
	115 mM NaCl	34.16 ± 2.52 ^bc^	38.29 ± 2.37 ^b^	30.22 ± 1.81 ^cd^	29.49 ± 2.19 ^de^	44.55 ± 3.52 ^a^
Total seeds per plant	0 mM NaCl	1589.00 ± 92.89 ^ab^	1604.92 ± 46.64 ^ab^	1521.50 ± 121.44 ^b^	1552.09 ± 73.64 ^ab^	1635.00 ± 20.52 ^a^
	115 mM NaCl	965.09 ± 22.45 ^d^	757.42 ± 17.39 ^e^	918.08 ± 44.38 ^d^	1066.33 ± 38.37 ^c^	748.92 ± 69.42 ^e^
Seed weight per plant	0 mM NaCl	39.54 ± 2.44 ^a^	37.37 ± 0.67 ^b^	37.32 ± 2.00 ^b^	37.39 ± 0.99 ^b^	41.32 ± 1.83 ^a^
	115 mM NaCl	24.77 ± 0.27 ^c^	19.15 ± 0.49 ^d^	23.30 ± 0.99 ^c^	23.35 ± 0.50 ^c^	16.84 ± 0.81 ^e^
1000–seed weight	0 mM NaCl	25.17 ± 0.31 ^a^	23.35 ± 0.59 ^cd^	24.87 ± 0.67 ^ab^	24.16 ± 0.67 ^bc^	24.95 ± 0.63 ^ab^
	115 mM NaCl	23.08 ± 0.98 ^de^	21.34 ± 0.27 ^g^	22.21 ± 0.36 ^f^	21.97 ± 0.53 ^fg^	22.27 ± 0.40 ^ef^

Values represent the average of four replicates. ANOVAs were performed, followed by comparisons of means using DMRTs. Different superscript letters indicate statistically significant differences in means at a *p*-value < 0.05.

## Data Availability

The data for this study are available from the corresponding author upon request.

## Supplementary Materials

The following supporting information can be downloaded at https://www.mdpi.com/article/10.3390/plants14060885/s1, Figure S1: Rice inflorescence when treated with 0 (A), 75 (B), 115 (C), and 150 (D) mM of NaCl at the booting stage for 12 days; Table S1: Multifactorial analysis; Table S2: The primers used for the qPCR and PCR.

## Abbreviations

The following abbreviations are used in this manuscript:

AAC	Apparent amylose content
*C_i_*	Intercellular CO_2_ concentration
CRI1	Carotenoid reflectance index 1
DAR	Days after recovery
DAS	Days after salt treatment
E	Transpiration rate
GBSSI	Granule-bound starch synthase I
*g_s_*	Stomatal conductance
NDVI	Normalized difference vegetation index
PI	Performance index
*Pn*	Net photosynthetic rate
PSI	Photosystem I
PSII	Photosystem II
RAG	Rapidly available glucose
RC	Reaction center
RS	Resistant starch
SAG	Slowly available glucose
SPAD	Leaf greenness
