# Supplementary material for: Effects of Salt Stress at the Booting Stage of Grain Development on Physiological Responses, Starch Properties, and Starch-Related Gene Expression in Rice (Oryza sativa L.)
Source: Plants (Basel). 2025 Mar 12;14(6):885. doi: 10.3390/plants14060885 (PMC11944574; doi:10.3390/plants14060885)
Supplement: Supplementary file 1 [file plants-14-00885-s001.zip › Supplementary Table S2.docx]

**Supplementary Table S2** The primers used for the qPCR and PCR.

| Primer pairs | LOCUS ID | Strand | Primer sequence | Length | Tm(℃) | Product size |
| --- | --- | --- | --- | --- | --- | --- |
| 1 | LOC_Os06g04200 | Forward | CATGAACGTCGTGTTCGTCGGC | 22 | 60.8 | 173 bp |
|  |  | Reverse | CAGCCACAACGCTGGTATCCCAA | 23 | 61.8 |  |
| 2 | LOC_Os01g65810 | Forward | GGCAAGATGATGAAGAAGAAGGCGA | 25 | 59.2 | 259 bp |
|  |  | Reverse | TGGTTTTGCGGTTAGGGGACGA | 22 | 61.1 |  |
| 3 | LOC_Os03g08020(*EF1α*) | Forward | TCACCTTGGCACCGGTTG | 18 | 58.4 | 127 bp |
|  |  | Reverse | ATGGTTGTGGAGACCTTC | 18 | 51.7 |  |
